# Implementation of RNA sequencing and array CGH in the diagnostic workflow of the AIEOP-BFM ALL 2017 trial on acute lymphoblastic leukemia

**DOI:** 10.1007/s00277-020-03953-3

**Published:** 2020-02-20

**Authors:** Maximilian Schieck, Jana Lentes, Kathrin Thomay, Winfried Hofmann, Yvonne Lisa Behrens, Maike Hagedorn, Juliane Ebersold, Colin F. Davenport, Grazia Fazio, Anja Möricke, Swantje Buchmann, Julia Alten, Gunnar Cario, Martin Schrappe, Anke Katharina Bergmann, Martin Stanulla, Doris Steinemann, Brigitte Schlegelberger, Giovanni Cazzaniga, Gudrun Göhring

**Affiliations:** 1 Department of Human Genetics, Hannover Medical School, Carl-Neuberg-Str. 1, 30625 Hannover, Germany; 2 Research Core Unit Genomics, Hannover Medical School, Hannover, Germany; 3 Centro Ricerca Tettamanti, Pediatric Clinic, University of Milano-Bicocca, Fondazione MBBM, Monza, Italy; 4 Department of Pediatrics, University Hospital Schleswig-Holstein, Campus Kiel, Kiel, Germany; 5 Pediatric Hematology and Oncology, Hannover Medical School, Hannover, Germany

**Keywords:** Acute lymphoblastic leukemia, Fusion transcript, Diagnostics, IKZF1plus, Risk stratification, RNA sequencing

## Abstract

Risk-adapted therapy has significantly contributed to improved survival rates in pediatric acute lymphoblastic leukemia (ALL) and reliable detection of chromosomal aberrations is mandatory for risk group stratification. This study evaluated the applicability of panel-based RNA sequencing and array CGH within the diagnostic workflow of the German study group of the international AIEOP-BFM ALL 2017 trial. In a consecutive cohort of 117 children with B cell precursor (BCP) ALL, array analysis identified twelve cases with an *IKZF1*^plus^ profile of gene deletions and one case of masked hypodiploidy. Genetic markers *BCR*-*ABL1* (*n* = 1), *ETV6*-*RUNX1* (*n* = 25), and rearrangements involving *KMT2A* (*n* = 3) or *TCF3* (n = 3) were assessed by established conventional techniques such as karyotyping, FISH, and RT-PCR. Comparison of these results with RNA sequencing analysis revealed overall consistency in *n*=115/117 cases, albeit with one undetected *AFF1*-*KMT2A* fusion in RNA sequencing and one undetected *ETV6*-*RUNX1* fusion in conventional analyses. The combined application of RNA sequencing, FISH, and CGH+SNP array reliably detected all genetic markers necessary for risk stratification and will be used as the diagnostic standard workflow for BCP-ALL patients enrolled in the AIEOP-BFM ALL 2017 study. Prospectively, consistent collection of genome-wide CGH+SNP array as well as RNA sequencing data will be a valuable source to elucidate new prognostic lesions beyond established markers of pediatric ALL. In this respect, RNA sequencing identified various gene fusions in up to half of the *IKZF1*^plus^ (*n* = 6/12) and B-other (*n* = 19/36) cases but not in cases with hyperdiploid karyotypes (*n* = 35). Among these fusions, this study reports several previously undescribed in frame *PAX5* fusions, including *PAX5-MYO1G* and *PAX5-NCOA6*.

**Electronic supplementary material:**

The online version of this article (10.1007/s00277-020-03953-3) contains supplementary material, which is available to authorized users.

## Introduction

Acute lymphoblastic leukemia (ALL) is the most frequent childhood malignancy with an assumed worldwide annual incidence of more than 50,000 cases [1, 2]. Fortunately, many clinical trials recently demonstrated 5-year survival rates above 90% in pediatric ALL, whereas only a few decades ago, survival rates were below 20% [1, 3]. The great improvement in prognosis is essentially a result of iterative cycles of long-term clinical trials. These trials helped to identify biologic subtypes of ALL, to assess their distinct response to treatment and risk of relapse, and to develop individual risk-adapted therapies [4].

Risk stratification within the recently completed AIEOP-BFM ALL 2009 trial, an international collaborative treatment protocol for children and adolescents with acute lymphoblastic leukemia, was based on immunophenotype, specific chromosomal aberrations, and response to treatment assessed by minimal residual disease (MRD). The two chief chromosomal aberrations relevant to risk stratification in AIEOP-BFM ALL 2009 were the *AFF1*-*KMT2A* gene fusion and a hypodiploid karyotype. The fusion *BCR*-*ABL1* represented an exclusion criterion and those cases were treated within the EsPhALL study [5]. Cases showing none of these genetic markers were treated based on their response to treatment and MRD has proven to be an effective measure to identify patients with a high risk of relapse [6]. However, it is known that other genetic aberrations, some of them recurrent, exist in the MRD high-risk group and great efforts are being made to identify and characterize these aberrations, as this knowledge will be key to improve risk stratification, to introduce targeted treatment, and hopefully to increase long-term survival rates [7]. Therefore, the diagnostic workflow of the current AIEOP-BFM ALL 2017 study investigates for an extended set of stratification relevant gene fusions (i.e., *ETV6*-*RUNX1*, *AFF1*-*KMT2A*, and *TCF3-HLF*) as well as the *IKZF1*^plus^ subtype [8, 9]. It is noteworthy that *IKZF1*^plus^ is not defined by a single genetic aberration, but by deletions in six different genetic loci (i.e., deletion of *IKZF1* complemented by at least one additional deletion in *CDKN2A*, *CDKN2B*, *PAX5*, or *CRLF2*-*P2RY8*, and no deletion in *ERG*) [8]. The *IKZF1*^plus^ profile of gene deletions points at the necessity that future routine diagnostic workflows, which are currently often limited to specific genes of interest using fluorescence in situ hybridization (FISH) and polymerase chain reaction (PCR) analyses, will have to assess a wider range of targets in order to detect novel and yet undefined profiles relevant to risk stratification.

We here report on our results from implementing analysis of the leukemia transcriptome using panel-based RNA sequencing and genome-wide copy-number analysis using array CGH into the diagnostic workflow of the German study group of the international AIEOP-BFM ALL 2017 trial. In addition to assessing detection of *IKZF1*^plus^ in array CGH, the major aim of this study was to compare the RNA-seq-based detection of above mentioned gene fusions with well-established techniques such as karyotyping, FISH, and PCR analyses. We also discuss expanded use of RNA-seq data beyond stratification relevant gene fusions, which in some cases identified potentially targetable lesions. Prospectively, the consistent collection of genome-wide CGH+SNP array as well as RNA-seq data within the AIEOP-BFM ALL 2017 trial is a valuable resource for elucidation of new prognostic markers of pediatric ALL.

## Methods

### Patients and samples

This study was performed on a consecutive cohort of patients with pediatric ALL sent to our genetic reference laboratory for initial cytogenetic and molecular genetic diagnostics. All patients were treated in German centers and were enrolled in the AIEOP-BFM ALL 2009 trial (EudraCT no. 2007-004270-43). Written informed consent was obtained from the legal representatives (and the patients, if applicable). The study cohort consisted of *n* = 117 patients (*n* = 65 male, *n* = 52 female) with a median age of 4.8 years. Of these patients, 6 (5.1%) were classified with an immunophenotype of pro-B-ALL (B-I), 72 (61.5%) with a common-ALL (B-II), and 39 (33.3%) with a pre-B-ALL (B-III).

Bone marrow (BM) aspirate was obtained at the time of initial diagnosis. BM from heparinized tubes was used for cytogenetic analyses (karyotyping and FISH). RNA was preferably isolated from EDTA BM, or if necessary from heparin BM, on a Chemagic 360 instrument as per the manufacturer’s instructions (PerkinElmer, Baesweiler, Germany). RNA integrity (RIN value) was measured by Bioanalyzer 2100 (Agilent Technologies, Waldbronn, Germany). Isolation of DNA from EDTA BM was performed using the QIAamp DNA Blood Midi Kit (Qiagen, Hilden, Germany).

### Genetic markers relevant for risk stratification

This study assisted to optimize the diagnostic standard workflow of the German study group of the AIEOP-BFM ALL 2017 trial. Using the methods listed below, the following genetic markers relevant for risk stratification were evaluated: gene fusions (*ETV6*-*RUNX1*, *AFF1*-*KMT2A*, and *TCF3-HLF*), hypodiploidy (< 45 chr.), and *IKZF1*^plus^. *BCR*-*ABL1* is an exclusion criterion for the AIEOP-BFM ALL trial, as these cases are treated within the EsPhALL study instead.

### Karyotyping and FISH analysis

For all patients, short-term cultures (24–48 h) were set up from BM aspirates. Chromosome preparation, fluorescence R-banding, and FISH were performed as previously described [10]. Karyotypes were described according to the International System for Human Cytogenetic Nomenclature (ISCN, 2016). In addition to classical banding analysis, the diagnostic standard protocol included FISH analyses for *BCR-ABL1* and *ETV6-RUNX1* fusions as well as for translocations affecting the genes *KMT2A* and *TCF3* (FISH probes: Vysis LSI *BCR*/*ABL* Dual Color, Abbott, Wiesbaden, Germany; Vysis LSI *ETV6*(*TEL*)/*RUNX1*(*AML1*) ES Dual Color, Abbott; Vysis LSI *MLL*(*KMT2A*) Dual Color, Abbott; SureFISH 19p13.3 *TCF3* DF, Agilent) [10].

### Reverse transcriptase PCR

Reverse transcriptase PCR (RT-PCR) was performed in parallel to cytogenetic analyses whenever EDTA-material was available (74/117 cases) for the qualitative detection of fusion transcripts of *BCR-ABL1*, *ETV6-RUNX1*, and *AFF1*-*KMT2A* as published elsewhere [11, 12]. In cases where only heparinized material was available, no RT-PCR was performed due to possible inhibitory effects of heparin on amplification. cDNA quality was verified by quantification of the *ABL1* gene.

### Panel-based RNA sequencing and data analysis

RNA-seq was performed using the TruSight RNA Pan-Cancer Panel (Illumina, San Diego, CA, USA) according to the manufacturer’s instruction. The panel targets 1385 cancer genes (see online Tab.S1) and detects fusions between genes targeted by the panel but also fusions of targeted genes with non-target genes. Prior to this study, the RNA-seq method was successfully validated on commercially available reference material (SeraSeq® Myeloid Fusion RNA Mix, Hiss Diagnostics GmbH, Freiburg, Germany), external reference samples with defined fusions provided by the group of Giovanni Cazzaniga (University of Milano-Bicocca, Fondazione MBBM, Monza, Italy), and genetically characterized cell lines and samples from our laboratory (see Tab.S2 for tested fusions). Gene-pseudogene “fusions” and recurrent technical artifacts were defined during this validation process and excluded from later data analysis. Each RNA-seq run contained a pooled cDNA library of eight patient samples and was executed on an Illumina MiSeq machine using MiSeq® Reagent Kit v3 (150 cycles) with a PE MiSeq® Flow Cell. The anticipated minimum number of reads per sample was 3 million.

Data analysis was performed using the Illumina BaseSpace apps TopHat Alignment (version 1.0.0, read mapping on hg19 reference genome by TopHat2 [13], fusion calling by TopHat-Fusion [14]) and RNA-seq Alignment (version 1.1.0, read mapping on hg19 reference genome by STAR [15], fusion calling by Manta [16]) using standard settings (https://basespace.illumina.com/apps). Fusion transcripts with a low number of split-reads (< 10) were excluded as likely false-positives, unless the fusion was verified with a second method. The turn-around time from RNA isolation to detection of fusion transcripts using this workflow is 4 days.

Validation of single gene fusions detected in RNA-seq was carried out using the HemaVision®-28N multiplex RT-PCR Kit if applicable and according to the manufacturer’s instructions (DNA Diagnostic, Risskov, Denmark).

### Array CGH

Array-based comparative genomic hybridization (array CGH) was performed using a 400 k SurePrint G3 Custom CGH Human Genome Microarray (e-Array design 84704, Agilent Technologies, Waldbronn, Germany) according to the manufacturer’s protocol by hybridizing 500 ng of patient DNA isolated from EDTA material against sex-matched human reference DNA (Megapool Reference DNA, Kreatech, Amsterdam, The Netherlands). Regions of genomic copy number change were analyzed and interpreted with Agilent Genomic Workbench 7.0 software under the following analysis setting: aberration detection algorithm ADM-2, threshold 6.0, aberration filter of at least four aberrant probes (resulting in an average genome-wide resolution of ~ 30 kb), and average log2 ratios of + 0.3 for gains and − 0.3 for losses.

The original description of the *IKZF1*^plus^ gene deletion profile by Stanulla et al. has been based on MLPA analyses targeting *IKZF1*, *CDKN2A*, *CDKN2B*, *PAX5*, and *CRLF2*-*P2RY8*, as well as multiplex PCR for the detection of *ERG* deletions. Prior to this study, reproducibility of *IKZF1*^plus^ profiles in our high-resolution array CGH was verified for selected cases (data not shown).

Cases indicative of masked hypodiploidy (i.e., presence of multiple tetrasomies) were analyzed on CGH+SNP array (e-Array design 85320, Agilent Technologies) using sex-matched reference DNA (Human Reference DNA, Agilent Technologies).

## Results

### Panel-based RNA-seq results are consistent with fusions detected by cytogenetic and RT-PCR analyses

Within the cohort of 117 consecutive patients with pediatric BCP-ALL, the following genetic markers relevant for inclusion/exclusion and risk stratification of patients were observed: *BCR-ABL1* fusion (*n* = 1; exclusion criterion and referral to EsPhALL trial), *ETV6-RUNX1* fusion (*n* = 26), *AFF1*-*KMT2A* fusion (*n* = 2), hypodiploidy (*n* = 1), and *IKZF1*^plus^ (*n* = 12). Other observed genetic markers not relevant for risk stratification within the AIEOP-BFM ALL 2017 trial were as follows: *TCF3* rearrangement (*n* = 3; *TCF3-PBX1* fusions), *KMT2A* rearrangement (*n* = 1; *KMT2A*-*MLLT1* fusion; aberration is only relevant for stratification in cases < 1 year of age), and hyperdiploid karyotype with more than 50 chromosomes (*n* = 35). The remaining *n* = 36 cases were classified as B-other due to absence of the above-mentioned genetic markers (Table 1).

**Table 1:** Characteristics of the cohort of 117 consecutive cases with pediatric BCP-ALL. Patients were enrolled to the AIEOP-BFM ALL 2009 trial at different participating clinics throughout Germany. The sex and age distribution of the total cohort was within the expected range of pediatric BCP-ALL [17, 18]. Genetic markers relevant for inclusion/exclusion of patients and risk stratification within the AIEOP-BFM ALL 2017 trial are listed in the upper part of the table. Array CGH identified the *IKZF1*^plus^ gene deletion profile in twelve cases (10%) and CGH+SNP array identified one case of masked hypodiploidy. Beyond *ETV6*-*RUNX1*, *BCR*-*ABL1*, *AFF1*-*KMT2A*, and *TCF3*-*PBX1* fusions, RNA-seq identified additional fusion transcripts in half of *IKZF1*^plus^ cases and in about half of B-other cases (details in Table 3). Furthermore, one case with a *KMT2A* split signal in FISH analysis could be specified as a *KMT2A*-*MLLT1* fusion by RNA-seq. *na*, not applicable due to small number of cases

	Genetic subtype	Number of cases	Ratio male/female	Median age years (min–max)
Relevant for risk stratification	*BCR-ABL1*	1 (1%)	na	na
	*ETV6-RUNX1*	26 (22%)	1.36	4.0 (1.6–17.9)
	*AFF1*-*KMT2A*	2 (2%)	na	na
	Hypodiploidy	1 (1%)	na	na
	*IKZF1*^plus^	12 (10%)	2.00	8.7 (2.5–17.0)
	*IKZF1*^plus^ with fusion in RNA-seq	6 (5%)	2.00	8.6 (2.5–17.0)
	*IKZF1*^plus^ without fusion in RNA-seq	6 (5%)	2.00	8.7 (3.6–15.2)
Not relevant for risk stratification	*TCF3* rearrangement	3 (3%)	na	na
	*KMT2A* rearrangement	1 (1%)	na	na
	B-other	36 (31%)	1.00	6.2 (1.2–17.4)
	B-other with fusion in RNA-seq	17 (15%)	0.70	4.2 (2.2–15.0)
	B-other without fusion in RNA-seq	19 (16%)	1.38	9.5 (1.2–17.4)
	Hyperdiploidy (> 50 chr.)	35 (30%)	1.19	4.2 (2.1–13.8)
	Total	117	1.25	4.8 (0.3–17.9)

Fusion detection by RNA-seq was highly consistent with results obtained from cytogenetic and RT-PCR analyses for 115 out of 117 analyzed cases. The two discordant cases were case 29, which was misclassified by RNA-seq, and case 26, which was misclassified in conventional analyses (see online Tab.S3 for a complete overview on all cases and results). In detail, case 29 presented in FISH analysis with a split of the *KMT2A* locus and subsequent analyses using FISH on interphase and metaphase nuclei as well as RT-PCR using the HemaVision-28N kit were able to identify this fusion to be derived from a small insertion of the 5′*KMT2A*-Locus into chromosome 4 (ins(4;11)). However, this cryptic *AFF1*-*KMT2A* fusion was not reported by either of the two Illumina BaseSpace apps, despite its existence in the raw RNA-seq reads. The dependency on accurate fusion calls by alignment algorithms was also indicated by other cases in this study. Case 15 tested positive for *ETV6*-*RUNX1* fusion in FISH and RT-PCR analyses, whereas only the RNA-seq Alignment app and not the TopHat Alignment app detected this fusion. In contrast, all nine cases with *P2RY8*-*CRLF2* fusions were detected by the TopHat Alignment app only (cases 55–63).

On the other side, conventional analyses also generated one false negative result in this study. FISH and regular RT-PCR indicated no *ETV6*-*RUNX1* fusion for case 26, whereas RNA-seq gave a positive result. Subsequently, the fusion of *ETV6*-*RUNX1* could be confirmed using the HemaVision-28N multiplex RT-PCR Kit. In detail, analysis of the RNA sequence revealed a fusion of *ETV6* exon 5 to *RUNX1* exon 3, which should have been detectable by routine RT-PCR primers and FISH analyses. Furthermore, case 16 showed an *ETV6-RUNX1* fusion in RNA-Seq and FISH resulting from a variant breakpoint in *ETV6* exon 4, which could not be detected via routine RT-PCR due to a loss of the primer binding site.

All other fusions addressed by karyogram, FISH, or RT-PCR were either confirmed (i.e., *BCR*-*ABL1*, *PBX1*-*TCF3*) or specified in more detail by RNA-seq, as was the case for the identification of *MLLT1* as the fusion partner of *KMT2A* (11q23.3-19p13.3; case 30).

### Array CGH identifies *IKZF1*^plus^ cases in B-other and hyperdiploid subtypes of BCP-ALL

All cases negative for a risk stratifying marker in cytogenetic and RT-PCR analyses were analyzed by array CGH for presence of the *IKZF1*^plus^ deletion profile. Under these conditions, a total of 84 cases qualified for array CGH (two of these cases had insufficient DNA from time point of diagnosis). The *IKZF1*^plus^ deletion profile was observed in twelve cases. With a median age of 8.7 compared with 4.8 years, the *IKZF1*^plus^ cases were older than the overall cohort (Table 1). In all cases, the mandatory deletion of *IKZF1* was accompanied by chromosome 9 deletions, whereas no interstitial deletions between *P2RY8* and *CRLF2* were observed (Table 2). The gene *IKZF1* was affected by small intragenic deletions, except for cases 33, 35, and 43. Various deletions could be observed on chromosome 9, ranging from small focal deletions up to losses of the entire chromosomal arm. At the conclusion of induction therapy, nine cases had a positive MRD result and would therefore qualify for the high-risk treatment group of the AIEOP-BFM ALL 2017 trial (Table 2).

**Table 2:** Cases of *IKZF1*^plus^ identified by array CGH. Cases negative for risk stratifying markers *BCR*-*ABL1*, *ETV6*-*RUNX1*, *AFF1*-*KMT2A*, *TCF3-HLF*, and hypodiploidy were analyzed by array CGH. The *IKZF1*^*plus*^ deletion profile was identified in a total of twelve cases. The mandatory deletion of *IKZF1* was accompanied by deletions on chromosome 9 in all cases, interstitial deletions between *P2RY8* and *CRLF2* were not observed. At the end of induction therapy, nine cases had a positive MRD result and would therefore qualify for the high-risk treatment group of the AIEOP-BFM ALL 2017 trial. *MRD pos*, patients with a positive/measurable MRD signal including low positive, not quantifiable signal (based on the EuroMRD Guidelines); *TP1*, conclusion of induction therapy on day 33

Patient data				*IKZF1*^plus^ profile						Risk stratification	
Case no.	Sex	Age at diagnosis	Immunophenotype	*IKZF1*	*CDKN2A*	*CDKN2B*	*PAX5*	*P2RY8-CRLF2*	*ERG*	MRD (TP1)	Suggested treatment group
32	m	17.0	common-ALL (B-II)	Deletion (7p12.2)	Normal	Normal	Deletion (9p13.2)	Normal	Normal	pos	High risk
33	m	13.1	common-ALL (B-II)	Deletion (7p14.3p12.2)	Deletion (9p21.3)		Deletion (9p13.2)	Normal	Normal	neg	Standard risk
34	m	5.6	common-ALL (B-II)	Deletion (7p12.2)	Deletion (9p21.3)		Deletion (9p13.2)	Normal	Normal	pos	High risk
35	f	2.5	pre-B-ALL (B-III)	Deletion (7p13p12.1)	Deletion (9p24.3p13.2)			Normal	Normal	neg	Standard risk
36	m	3.2	common-ALL (B-II)	Deletion (7p12.2)	Deletion (9p24.3p13.2)			Normal	Normal	pos	High risk
37	f	11.6	common-ALL (B-II)	Deletion (7p12.2)	Deletion (9p22.1p13.1)			Normal	Normal	pos	High risk
38	m	3.6	pre-B-ALL (B-III)	Deletion (7p12.2)	Deletion (9p24.3p13.1)			Normal	Gain	pos	High risk
39	m	3.9	common-ALL (B-II)	Deletion (7p12.2)	Deletion (9p24.3p13.2)			Normal	Normal	pos	High risk
40	f	12.2	common-ALL (B-II)	Deletion (7p12.2)	Deletion (9p21.3p13.2)		Normal	Normal	Normal	pos	High risk
41	m	15.2	common-ALL (B-II)	Deletion (7p12.2)	Normal	Normal	Deletion (9p13.2)	Normal	Normal	pos	High risk
42	m	7.4	common-ALL (B-II)	Deletion (7p12.2)	Deletion (9p21.3p21.2)		Gain	Gain	Gain	pos	High risk
43	f	9.9	pre-B-ALL (B-III)	Deletion (7p22.3q36.3)	Deletion (9p21.3p13.1)			Gain	Gain	neg	Standard risk

### RNA-seq detects gene fusions in the *IKZF1*^plus^ and B-other subtypes but not in hyperdiploid BCP-ALL

As expected, RNA-seq also identified fusions between genes not covered by the diagnostic standard protocol of the AIEOP-BFM ALL 2017 trial. No fusions were detected in the large group of hyperdiploid cases, while fusion transcripts were detected at a high frequency in up to half of the cases in subtypes *IKZF1*^plus^ (*n* = 6/12 cases) and B-other (*n* = 17/36 cases, Table 1). Of these 23 cases, only 13 presented an aberrant karyotype in banding analysis (online Tab.S3).

Cases with an aberrant karyotype displayed the following fusions: *ETV6-NTRK3* (12p13.2-15q25.3), *PAX5-NCOA6* (9p13.2-20q11.22), *TERF2-JAK2* (16q22.1-9p24.1), *HOOK3-FGFR1* (8p11.21-8p11.23), *MEF2D-BCL9* (1q22-1q21.2), *PAX5-DACH1* (9p13.2-13q21.33), *PAX5-ETV6* (9p13.2-12p13.2), *PAX5-LINC01251* (9p13.2-9p13.3), *ZNF618-NUTM1* (9q32-15q14), and *P2RY8-CRLF2* (*n* = 5 cases, Xp22.33 and Yp11.2). Notably, all six cases with a structurally aberrant chromosome 9p showed a gene fusion involving the genes *JAK2* (*n* = 1) or *PAX5* (*n* = 5). Fusion transcripts found in patients with a completely normal karyotype and no other markers were *EBF1-PDGFRB* (5q33.3-5q32, case 32), *PAX5-MYO1G* (9p13.2-7p13, case 35), and *P2RY8-CRLF2* (cases 59 and 62). Fusions of *ETV6*-*IKZF1* (12p13.2-7p12.2) and *P2RY8*-*CRLF2* were found in two cases with normal karyotypes at a reduced level of validity (due to poor morphology and limited number of metaphases; cases 33 and 57). No metaphases were available for case 47 (fusions *ETV6*-*IKZF1* and *IKZF1*-*DLG2* (7p12.2-11q14.1)), case 48 (*EP300*-*ZNF384* (22q13.2-12p13.31) fusion), and cases 56 and 60 (both *P2RY8*-*CRLF2* fusion). Presence of all fusions, except for *TERF2*-*JAK2*, *IKZF1*-*DLG2*, and *HOOK3*-*FGFR1*, could be indirectly confirmed by detection of an array CGH aberration in at least one of the two fusion genes (i.e., intronic breakpoint corresponding to the RNA-seq spanning read, Table 3). Further validation of RNA-seq fusions, including *TERF2*-*JAK2* and *HOOK3*-*FGFR1*, was achieved by FISH results (i.e., split signal involving the corresponding gene loci), and in most cases in banding analysis (i.e., identification of respective chromosome translocations, or at least of respective aberrant chromosomes, online Tab.S3).

**Table 3:** RNA-seq results for genes not targeted by the diagnostic standard protocol using FISH or RT-PCR analysis. Additional gene fusions were detected in the *IKZF1*^plus^ and B-other subtypes but not in hyperdiploid BCP-ALL. Fusions were detected in cases with aberrant but also with normal karyotypes as assessed by R-banding and FISH. The 5′-3′ orientation (➔) of fused coding gene regions to one another is indicated according to the spanning read observed in TopHat alignment (details in Tab.S3). Information on potential in frame fusions of the coding sequences (CDS) is based on TopHat spanning reads as well. Presence of all fusions, except *IKZF1*-*DLG2*, could be validated indirectly by array CGH, FISH, or retrospective banding analysis. Previous reports on fusion genes observed in this study are summarized

Genetic subtype	Patient data				RNA-seq			Previous report on fusion gene
	Case no.	Sex	Age at diagnosis	Immunophenotype	Fusion	Orientation of fusion partners (observed CDS)	Corresponding aberration in aCGH (affected genes)	
***IKZF1***^**plus**^	32	m	17.0	common-ALL (B-II)	***EBF1-PDGFRB***	➔➔ (in and out of frame fusions)	pos (*EBF1* and *PDGFRB*)	Roberts et al., 2014, *NEJM*
	33	m	13.1	common-ALL (B-II)	***ETV6-IKZF1***	➔➔ (out of frame fusion)	pos (*IKZF1*)	no previous report
	34	m	5.6	common-ALL (B-II)	***ETV6-NTRK3***	➔➔ (in frame fusion)	pos (*ETV6* and *NTRK3*)	Roberts et al., 2014, *NEJM*
	35	f	2.5	pre-B-ALL (B-III)	***PAX5-MYO1G***	➔➔ (in and out of frame fusions)	pos (*PAX5* and *MYO1G*)	no previous report
	36	m	3.2	common-ALL (B-II)	***PAX5-NCOA6***	➔➔ (in frame fusion)	pos (*PAX5* and *NCOA6*)	no previous report
	37	f	11.6	common-ALL (B-II)	***TERF2-JAK2 JAK2-TERF2***	➔➔ (in frame fusions)	neg	Roberts et al., 2014, *NEJM*
**B-other**	47	m	2.4	pro-B-ALL (B-I)	***ETV6-IKZF1 IKZF1-DLG2***	➔➔ (out of frame fusions)	pos (*ETV6*)	no previous report
	48	f	4.2	pro-B-ALL (B-I)	***EP300-ZNF384***	➔➔ (in frame fusion)	pos (*EP300*)	Gocho et al., 2015, *Leukemia*
	49	m	13.7	common-ALL (B-II)	***HOOK3-FGFR1***	➔➔ (in frame fusion)	neg	Reshmi et al., 2017, *Blood*
	50	f	12.9	common-ALL (B-II)	***MEF2D-BCL9***	➔➔ (in frame fusions)	pos (*MEF2D* and *BCL9*)	Gu et al., 2016, *Nat Commun*
	51	m	3.7	common-ALL (B-II)	***PAX5-DACH1***	➔➔ (in frame fusion)	pos (*PAX5* and *DACH1*)	Nebral et al., 2009, *Leukemia*
	52	f	15.0	pre-B-ALL (B-III)	***PAX5-ETV6***	➔➔ (fusion with non-coding *ETV6* exon)	pos (*PAX5* and *ETV6*)	Cazzaniga et al., 2001, *Cancer Res*
	53	f	6.5	common-ALL (B-II)	***PAX5-LINC01251***	➔➔ (fusion with non-coding RNA)	pos (*PAX5* and *LINC01251*)	no previous report
	54	f	8.1	pre-B-ALL (B-III)	***ZNF618-NUTM1***	➔➔ (in frame fusion)	pos (*NUTM1*)	Gu et al., 2019, *Nat Genet*
	55	f	2.2	pre-B-ALL (B-III)	***P2RY8-CRLF2***	➔➔ (fusion of *P2RY8* non-coding exon 1 to *CRLF2* coding region)	pos (*P2RY8* and *CRLF2*)	Russell et al., 2009, *Blood*
	56	f	3.1	common-ALL (B-II)	***P2RY8-CRLF2***	➔➔ (fusion of *P2RY8* non-coding exon 1 to *CRLF2* coding region)	pos (*P2RY8* and *CRLF2*)	Russell et al., 2009, *Blood*
	57	f	3.2	common-ALL (B-II)	***P2RY8-CRLF2***	➔➔ (fusion of *P2RY8* non-coding exon 1 to *CRLF2* coding region)	pos (*P2RY8* and *CRLF2*)	Russell et al., 2009, *Blood*
	58	m	3.2	common-ALL (B-II)	***P2RY8-CRLF2***	➔➔ (fusion of *P2RY8* non-coding exon 1 to *CRLF2* coding region)	pos (*P2RY8* and *CRLF2*)	Russell et al., 2009, *Blood*
	59	m	3.4	common-ALL (B-II)	***P2RY8-CRLF2***	➔➔ (fusion of *P2RY8* non-coding exon 1 to *CRLF2* coding region)	pos (*P2RY8* and *CRLF2*)	Russell et al., 2009, *Blood*
	60	f	4.2	pre-B-ALL (B-III)	***P2RY8-CRLF2***	➔➔ (fusion of *P2RY8* non-coding exon 1 to *CRLF2* coding region)	pos (*P2RY8* and *CRLF2*)	Russell et al., 2009, *Blood*
	61	f	5.6	pre-B-ALL (B-III)	***P2RY8-CRLF2***	➔➔ (fusion of *P2RY8* non-coding exon 1 to *CRLF2* coding region)	pos (*P2RY8* and *CRLF2*)	Russell et al., 2009, *Blood*
	62	m	10.3	common-ALL (B-II)	***P2RY8-CRLF2***	➔➔ (fusion of *P2RY8* non-coding exon 1 to *CRLF2* coding region)	pos (*P2RY8* and *CRLF2*)	Russell et al., 2009, *Blood*
	63	m	7.3	common-ALL (B-II)	***P2RY8-CRLF2***	➔➔ (fusion of *P2RY8 *non-coding exon 1 to *CRLF2* coding region)	np	Russell et al., 2009, *Blood*

## Discussion

This study aimed to evaluate the applicability of panel-based RNA-seq and array CGH for detecting relevant gene fusions as well as chromosomal deletions for risk stratification of pediatric BCP-ALL patients. This knowledge is intended to support optimization of the diagnostic workflow of the German study group of the international AIEOP-BFM ALL 2017 trial by reducing the number of necessary diagnostic tests, while assuring robustness of aberration detection. As discussed later, the here presented results conclusively show that panel-based RNA-seq and CGH+SNP array are powerful tools for the detection of fusion genes and genome-wide copy number aberrations. However, these techniques still need to be supported by FISH analysis in order to guarantee reliable detection of relevant fusions for all cases. The second and more broadly defined aim of this study was to examine the detection of genetic lesions beyond established markers, as recent publications have demonstrated that patients of the B-other subtype frequently harbor fusion transcripts of possible therapeutic relevance [19–21]. Prospectively, successful integration of genome-wide CGH+SNP array and RNA-seq targeting almost 1385 transcripts into the AIEOP-BFM ALL 2017 diagnostic workflow could help to generate the data essential for revealing new genetic markers of pediatric ALL in the future.

The *IKZF1*^plus^ subtype itself is a good example for a new risk marker, which has been identified due to simultaneous consideration of multiple genetic loci. Notably, without analysis for *IKZF1*^plus^, ten cases of this study would have been assigned as B-other and two cases as hyperdiploid ALL, with no further genetic risk stratification in both groups. One needs to be aware that occasional *IKZF1*^plus^ profiles might be present in cases not tested by array CGH in our workflow (e.g., *ETV6-RUNX1*). Projected onto the whole cohort of 117 BCP-ALLs, we therefore observed *IKZF1*^plus^ in at least 10% of the cases, which is higher than the originally published share of 6% by Stanulla et al. [8]. Use of array CGH instead of MLPA and PCR-based analysis does not seem to be the reason for this discrepancy, as all deletions relevant for of *IKZF1*^plus^ (Tab.S4) would be detectable by MLPA analyses as described by Stanulla et al. Furthermore, the *ERG* locus is covered by 37 array CGH probes, of which seven probes are located in the region assessed by *ERG* deletion PCR in Stanulla et al. Despite the satisfactory probe coverage of *ERG* in array CGH, the higher sensitivity of PCR for deletion detection needs to be acknowledged, which might influence *IKZF1*^plus^ detection. However, the high number of *IKZF1*^plus^ cases in our study is more likely to be explained by the small cohort size in our analysis as our extended array CGH data set on more than 600 cases comprises a lower number of *IKZF1*^plus^ within the B-other and hyperdiploid cases (data not shown). In line with Stanulla et al., the age of onset of *IKZF1*^plus^ cases was considerably higher than observed in the complete cohort (median age 8.7 vs 4.8 years, Table 1). It is important to mention that in the AIEOP-BFM ALL 2017 trial, the final risk stratification within *IKZF1*^plus^ is dependent on MRD-levels and not all cases with an *IKZF1*^plus^ deletion profile receive high-risk treatment. Within our cohort, nine of the twelve *IKZF1*^plus^ cases were MRD positive or inconclusive on treatment day 33 and therefore would be assigned to the high-risk treatment group of the AIEOP-BFM ALL 2017 trial.

In summary, array CGH analysis is a well-established method in many laboratories and in our study, its implementation in the diagnostic workflow was successful without difficulty. One important limitation of the method is the amount of leukemic blasts in BM aspirate obtained at the time of diagnosis, which should be at least 20% in order to reliably detect leukemia-associated genetic aberrations. On the other hand, CGH+SNP array proved to be more sensitive in detecting hyperdiploidy, which is in line with the previous observation that hyperdiploid leukemic cells frequently fail to proliferate in culture, thus resulting in an absence of aneuploidy in analyzed metaphases or deficiency of metaphases in general (Tab.S3 cases 83, 85, 90, 91, 94, 116) [22]. Furthermore, array analysis identified other highly relevant genetic aberrations beyond *IKZF1*^plus^, e.g., masked hypodiploidy and aberrations in line with fusions observed in RNA-seq analysis. For these reasons, copy number analysis using CGH+SNP arrays will be a central diagnostic tool in the AIEOP-BFM ALL 2017 trial to detect the current stratification relevant markers as well as to generate high-resolution genome-wide data for the definition of future genetic markers.

Detection of fusion genes using RNA-seq is a comparably new approach and not yet established in routine diagnostics for most malignancies. Our data demonstrate consistent results between RNA-seq and our routine diagnostic methods (karyogram, FISH, and RT PCR analyses). Considering the stratification relevant aberrations *BCR-ABL1* and *ETV6-RUNX1* as well as rearrangements involving either *KMT2A* or *TCF3*, which are relevant for stratification if resulting in an *AFF1*-*KMT2A* or *TCF3*-*HLF* fusion, only two discrepant results were observed (cases 26 and 29). For case 26, RNA-seq outperformed the routine FISH and RT-PCR methods by identifying an *ETV6*-*RUNX1* fusion, which could later be confirmed with a second RT-PCR kit. The reason for the failure of the routine methods to detect this fusion is still unclear. Case 29, with a small insertion of *KMT2A* into the *AFF1* gene, was the only example of a clear failure in fusion detection by RNA-seq. Even though present as multiple, identical reads at the RNA-seq raw data level, both applied alignment algorithms did not report the *AFF1*-*KMT2A* fusion, as they require the presence of at least three non-identical split reads for fusion calling. In our study, these two discrepant cases are the only two examples for false negative results. However, further influences exist, which can cause relevant fusions to remain undetected. These include a low percentage of leukemic blasts, loss of blasts during cell culture, cryptic or variant translocations, poor RNA quality, and notably the sensitivity of bioinformatics algorithms. Importantly, not all methods (karyogram, FISH, RT-PCR, and RNA-seq) are equally affected by these influences and a combination of methods would be able to rectify their respective weaknesses in most cases. Therefore, the diagnostic workflow of the German study group of AIEOP-BFM ALL 2017 trial will use FISH and panel-based RNA-seq as standard techniques for the detection of fusion genes. Karyotyping and RT-PCR will still be available but restricted to inconclusive cases only.

As a technical prerequisite, both alignment tools tested in this study will be applied in future RNA-seq analyses, as some fusions, like *P2RY8*-*CRLF2*, were detected by one of the algorithms only. Furthermore, the minimum number of sequencing reads per sample will be set to 3 million reads, even though our current data indicate that some fusion transcripts, like *ETV6-RUNX1*, are detectable at coverages below 2 million reads. However, a higher number of reads per sample will also facilitate detection of lowly expressed fusion genes as well as detection of fusions in samples with low blast counts. We detected no correlation between RNA-seq coverage and RIN value or *ABL1* copy numbers in RT-PCR analysis (data not shown). Thus, variance in RNA-seq coverage in our study might rather be a result of differences in library preparation and quantification, which can possibly be addressed by standardization and automatization of RNA-seq protocols. Another important technical aspect of our study is that RNA-seq was conducted on RNA isolated from BM in EDTA tubes sent to our laboratory within 24 h. Our results indicate that use of RNA stabilizing solutions, at least for the fusion genes of interest, seems expendable.

It needs to be acknowledged that this workflow, despite panel-based RNA-seq and genome-wide array CGH, will not detect all aberrations present in the here defined B-other group. For example, fusions affecting the *IGH* locus as well as translocations resulting in promoter or enhancer rearrangements (e.g., translocations of the *MYC* or *MECOM* locus) cannot be detected and these aberrations are being followed up on by FISH analysis on a research basis for selected cases. On the same note and in line with previous reports, our RNA-seq analysis detected additional fusion transcripts only in the B-other subtype, including cases 32–37, which all would have been deemed B-other until recently if not stratified as *IKZF1*^plus^ in our study [20, 21]. Interestingly, the previously described “targetable” kinase fusions *EBF1-PDGFRB*, *ETV6-NTRK3*, and *TERF2-JAK2* were all observed within the *IKZF1*^plus^ subtype [19, 23, 24]. Other detected fusions may provide prognostic information (*P2RY8-CRLF2*, *MEF2D-BCL9*) [25–27]. Many fusion transcripts have already been described in pediatric ALL (e.g., *HOOK3-FGFR1*, *PAX5-DACH1*, *EP300-ZNF384*, *PAX5*-*ETV6*, or *ZNF618*-*NUTM1*), even though their potential therapeutic or prognostic impact remains to be addressed in more detail [28–33]. To the best of our knowledge, some fusions identified in this study have not been described in the literature yet. A more detailed follow-up on the relevance of these fusions will be necessary, as our data on orientation and on in frame fusions of the coding sequences (CDS) gives only a first perception on potential functionality. Based on this limited data, *PAX5-MYO1G*, *PAX5-NCOA6*, and possibly *PAX5-LINC01251* seem to be promising new fusions, whose influence on the course of the disease warrants further consideration [34, 35]. Just recently, the clinical implication of chimeric in frame *PAX5* fusions has been highlighted, as well as the potentially initiating, subtype-defining character of *PAX5* alterations in B-ALL [33]. In this respect, particularly the fusions with *MYO1G* and *NCOA6*, first identified in this study, appear to leave the paired box DNA-binding domain of *PAX5* operational.

In conclusion, this study demonstrated that panel-based RNA-seq, supplemented by FISH analysis, and CGH+SNP arrays are a reliable combination of methods to detect the genetic markers *BCR*-*ABL1*, *ETV6*-*RUNX1*, hypodiploidy, *IKZF1*^plus^ as well as rearrangements of *KMT2A* and *TCF3* necessary for risk stratification in pediatric BCP-ALL. Foremost, acquisition of genome-wide copy number and panel-wide fusion data within the standard diagnostic workflow is expected to support identification of new genetic markers relevant for treatment in the future.

## Electronic supplementary material

ESM 1 (XLSX 64 kb)

## Abbreviations

ALL: Acute lymphoblastic leukemia
AIEOP-BFM ALL: International collaborative treatment protocol for children and adolescents with acute lymphoblastic leukemia
array CGH: Array-based comparative genomic hybridization
B-I: B-lineage immunophenotype pro-B-ALL
B-II: B-lineage immunophenotype common-ALL
B-III: B-lineage immunophenotype pre-B-ALL
BCP-ALL: B cell precursor acute lymphoblastic leukemia
BM: Bone marrow
B-other: Subtype of cases that tested negative for genetic markers relevant for AIEOP-BFM ALL 2017
CDS: Coding sequence
FISH: Fluorescence in situ hybridization
*IKZF1*^plus^: Profile of gene deletions relevant for risk stratification within AIEOP-BFM ALL 2017
ISCN: International System for Human Cytogenetic Nomenclature
MRD: Minimal residual disease
RNA-seq: RNA sequencing
RT-PCR: Reverse transcriptase polymerase chain reaction
